# Clinical implementation of artificial intelligence in neuroradiology with development of a novel workflow-efficient picture archiving and communication system-based automated brain tumor segmentation and radiomic feature extraction

**DOI:** 10.3389/fnins.2022.860208

**Published:** 2022-10-13

**Authors:** Mariam Aboian, Khaled Bousabarah, Eve Kazarian, Tal Zeevi, Wolfgang Holler, Sara Merkaj, Gabriel Cassinelli Petersen, Ryan Bahar, Harry Subramanian, Pranay Sunku, Elizabeth Schrickel, Jitendra Bhawnani, Mathew Zawalich, Amit Mahajan, Ajay Malhotra, Sam Payabvash, Irena Tocino, MingDe Lin, Malte Westerhoff

**Affiliations:** ^1^Department of Radiology and Biomedical Imaging, Brain Tumor Research Group, Yale School of Medicine, Yale University, New Haven, CT, United States; ^2^Visage Imaging, GmbH, Berlin, Germany; ^3^Department of Radiology and Biomedical Imaging, Yale School of Medicine, Yale University, New Haven, CT, United States; ^4^Department of Radiology, Yale University and Visage Imaging, New Haven, CT, United States

**Keywords:** artificial intelligence (AL), machine learning (ML), PACS (picture archiving and communication system), brain tumor, segmentation, feature extraction, glioma, BraTS

## Abstract

**Purpose:**

Personalized interpretation of medical images is critical for optimum patient care, but current tools available to physicians to perform quantitative analysis of patient’s medical images in real time are significantly limited. In this work, we describe a novel platform within PACS for volumetric analysis of images and thus development of large expert annotated datasets in parallel with radiologist performing the reading that are critically needed for development of clinically meaningful AI algorithms. Specifically, we implemented a deep learning-based algorithm for automated brain tumor segmentation and radiomics extraction, and embedded it into PACS to accelerate a supervised, end-to- end workflow for image annotation and radiomic feature extraction.

**Materials and methods:**

An algorithm was trained to segment whole primary brain tumors on FLAIR images from multi-institutional glioma BraTS 2021 dataset. Algorithm was validated using internal dataset from Yale New Haven Health (YHHH) and compared (by Dice similarity coefficient [DSC]) to radiologist manual segmentation. A UNETR deep-learning was embedded into Visage 7 (Visage Imaging, Inc., San Diego, CA, United States) diagnostic workstation. The automatically segmented brain tumor was pliable for manual modification. PyRadiomics (Harvard Medical School, Boston, MA) was natively embedded into Visage 7 for feature extraction from the brain tumor segmentations.

**Results:**

UNETR brain tumor segmentation took on average 4 s and the median DSC was 86%, which is similar to published literature but lower than the RSNA ASNR MICCAI BRATS challenge 2021. Finally, extraction of 106 radiomic features within PACS took on average 5.8 ± 0.01 s. The extracted radiomic features did not vary over time of extraction or whether they were extracted within PACS or outside of PACS. The ability to perform segmentation and feature extraction before radiologist opens the study was made available in the workflow. Opening the study in PACS, allows the radiologists to verify the segmentation and thus annotate the study.

**Conclusion:**

Integration of image processing algorithms for tumor auto-segmentation and feature extraction into PACS allows curation of large datasets of annotated medical images and can accelerate translation of research into development of personalized medicine applications in the clinic. The ability to use familiar clinical tools to revise the AI segmentations and natively embedding the segmentation and radiomic feature extraction tools on the diagnostic workstation accelerates the process to generate ground-truth data.

## Introduction

Artificial intelligence (AI) is a powerful technology that has significant potential impact in the development of personalized medicine solutions in medical imaging, but only a few of these algorithms are readily available in standard clinical practice, regardless of whether they are FDA approved (ACR Data Science Institute, n.d.; Benjamens et al., 2020; Garderen et al., 2021). One of the major limitations in successful translation of AI algorithms into clinical practice is the lack of multicenter annotated datasets that allow sufficient training of the algorithm to be for clinical translation. Solutions to this problem include data sharing agreements, development of image databank consortiums (MIDRC, TCIA, BraTS), and federated learning (MIDRC, n.d.; Clark et al., 2013; Menze et al., 2015; Bakas et al., 2017a,b, 2018; Sheller et al., 2020). While these methods provide important advances for algorithm development, the need for building large databases of individual hospital data for algorithm translation to specific patient population that the hospital serves still remains.

While one can retrospectively curate these databases, a more workflow efficient method is to develop these databases in real time. That is, to have a platform for clinically incorporated tools that allow building of large radiologist annotated clinical datasets. Several studies evaluated methods outside of clinical PACS that provide potential solutions for incorporation of ML into clinical practice, with some of the studies demonstrating role of ML in changing clinical decisions such as administration of contrast (Combès et al., 2021; Garderen et al., 2021; Thakur et al., 2022). These approaches are early adapters of ML in clinical practice and are demonstrating potential for future approaches.

Deep learning algorithms have proven themselves to be effective in various aspects of glioma imaging, including tumor segmentation and classifying tumor grade and genetic subtypes in gliomas. Central nervous system (CNS) malignancy is expected to be diagnosed in 24,530 patients this year, with 18,600 patients dying from this disease (Siegel et al., 2020, 2021). The most common CNS malignancy is glioma, which are clinicopathologically graded as glioblastoma (IDH wildtype, Grade 4), astrocytoma (IDH-mutant grade 4, 3, 2) according to the World Health Organization 2021 (WHO) grading scale (Louis et al., 2021). The glioma grade represents the overall malignant potential of the tumor and can be determined based on pathologic evaluation and molecular analysis, with most recent classification describing significant changes in 2021 (Gonzalez Castro and Wesseling, 2020; Louis et al., 2021; Rushing, 2021). While glioblastomas are the most aggressive tumor type with a median survival of 14.6 months after treatment, grade 1-3 gliomas and IDH-mutant grade 4 astrocytomas have better outcomes with mean survival times ranging 2–8 years (Claus et al., 2015; Liang et al., 2020; Poon et al., 2020). Molecular subtypes play an important role in response to treatment and overall survival. For instance, MGMT mutation in GBM can improve treatment response to alkylating agents (Hegi et al., 2005). In addition, IDH mutation is an important prognostic factor for patients with improved survival rates compared to IDH wildtype glioblastomas (Xia et al., 2015; Chen et al., 2016).

Brain segmentation and radiomic feature extraction tools have shown promising results for incorporation in clinical practice in the field of neuro-oncology, but clinical translation has been significantly hampered due to limited available annotated datasets and decreased performance of algorithms on geographically distinct validation datasets (Subramanian et al., 2021; Cassinelli Petersen et al., 2022; Jekel et al., 2022).

Systematic reviews on application of Machine Learning in Gliomas identified 695 studies on this area, however, none of them described routine clinical implementation of these tools (Subramanian et al., 2021; Cassinelli Petersen et al., 2022; Jekel et al., 2022). While these tools have shown promising results in tumor segmentation and generation of high throughput quantitative data, several challenges are preventing their integration into clinical practice (Rudie et al., 2019). These challenges include incorporation of these tools into clinical workflow, making them user-friendly to radiologists, making the algorithm assessment of tumors time efficient, and coordinating the software output with radiology report software. To incorporate glioma segmentation tools into existing clinical workflows, we developed automated and efficient tumor segmentation and feature extraction tools and integrated them into PACS that is used at our hospital system, thus connecting an inference system which handles calls from the PACS, and presents the segmentation results to the attending radiologists in an easily accessible manner within a suitable hanging protocol. This method has potential to accelerate clinical implementation of ML by incorporating the ML algorithm into the software that is used by radiologists. This approach also has potential for development of large annotated datasets during real-time clinical practice.

## Materials and methods

After IRB approval, pre-operative images were obtained from the Yale New Haven Hospital database in adult patients with pathologically proven gliomas grade 3 and 4. The MR imaging was performed before surgical diagnosis of these patients. The images were reviewed retrospectively and the patients were grouped into high and low grade glioma. The DICOM data was transferred, and automatically anonymized, from our clinical PACS to our research PACS (Visage AI Accelerator, Visage Imaging, Inc., San Diego, CA, United States) (Lin, 2020). On the research PACS, 3D segmentation of the whole tumor with surrounding edema was performed manually by a board certified neuroradiologist (MSA) who was blinded to the glioma grading and outcome (Supplementary Figure 1). This manual segmentation served as the reference standard to which the AI based automatic segmentation tool was tested. Specifically, an algorithm was trained to delineate the whole glioma portion on MRI FLAIR images. The AI network was trained using the BraTS 2021 dataset, which contains multi-institutional and multi-sequence MRI scans of gliomas (Menze et al., 2015; Bakas et al., 2017a,b). The algorithm was subsequently trained on an internal dataset from Yale New Haven Health. For a deep-learning network architecture we chose UNET Transformer (UNETR), which uses a transformer in the encoder instead of a classical convolutional layer because this has improved performance compared to other architectures for this task (Hatamizadeh et al., 2022). The algorithm was trained using volumetric patches of size 128 × 128 × 64 with a spacing of 1.5 cm^3^ × 1.5 cm^3^ × 2 cm^3^ using trilinear interpolation with sampling of the patches (Benjamens et al., 2020; ACR Data Science Institute, n.d.). All preprocessing was performed once per volume and this intermediary data was cached to use for training.

The UNETR has a larger VRAM consumption than the NNUNET. The NNUNET samples a patch with size 128 × 128 × 128 from the 1 mm^3^ × 1 mm^3^ × 1 mm^3^ image resulting in an effective size of cropped volume of 128 mm^3^, which would not fit into the available VRAM (24GB, NVIDIA TITAN RTX). To achieve a similar volume, we resampled in the z-direction from 1 to 2, which allowed us to reduce the patch size to 128 × 128 × 64. The automatically segmented brain tumor was pliable for manual modification using the existing segmentation tools available in Visage 7.

**FIGURE 1:**
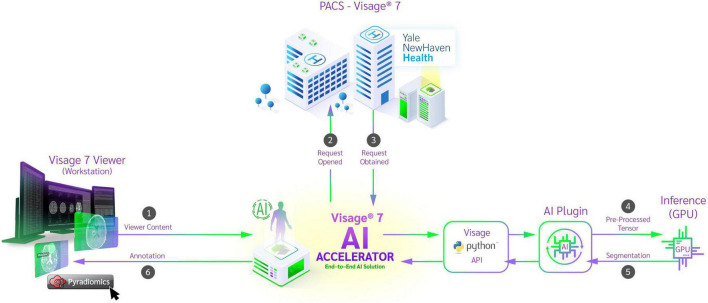
Schematic diagram of auto-segmentation and feature extraction tools incorporated into clinical PACS on Visage Imaging platform. From the workstation, the viewer content is passed to the Python API and the corresponding image data is retrieved from the PACS system. After preprocessing, a request is sent to the inference platform and a segmentation is returned. The segmentation is added to the study by the Python API and the result is displayed in the workstation. After reviewing the segmentation, the physician now has the ability to run radiomic feature extraction on the curated dataset.

The trained AI network was natively embedded using Docker and the NVIDIA Triton inference platform. The Docker container has access to the current protocol content and processes the data retrieved from the PACS. To ensure that the developed algorithm is deployed in a safe manner which is crucial in a clinical environment we adhered to the standards described by the Open Web Application Security Project (OWASP) (GitHub, Inc., n.d.). Their checklist includes good practices to secure Docker containers such as frequent updates, not exposing the docker daemon, limit user permissions and resources usage etc. (OWASP Cheat Sheet Series, n.d.). For preprocessing, brain extraction was performed (Isensee et al., 2019), the cropped data was reoriented, resampled using trilinear interpolation and the voxel intensities normalized using z-score normalization. The preprocessed image data was then passed to the inference platform through an HTTP request. The platform utilized mixed precision and TensorRT to boost inference speed. Inference was performed using a sliding window and test-time-augmentation in the form of flipping along each axis. The result was then passed back to the Docker container which converted the results to an overlay visible in Visage 7 (Figure 1). To reduce the burden on the radiologist in these tasks, several improvements were implemented into the Visage PACS client (Visage 7-1-18). These included a Quad multi-planar reformat comparison layout with 3D registration (automatic viewer registration tool) to facilitate viewing and segmentation. Given that certain aspects of the tumor are best segmented in specific MR sequences, it was critical to develop the function to copy/paste segmentation masks across MR sequences and ensure that the exact segmentation is transposed.

The resulting segmentation can be saved in PACS as a DICOM presentation state.

In a second step, we natively embedded PyRadiomics (Harvard Medical School, Boston, MA, United States) for feature extraction from those segmentations (Griethuysen et al., 2017). The algorithm utilizes both automatically and manually delineated structures present in the viewer content and calculates their radiomic features. Upon completion, the results are available to the researcher in JSON format, which can be stored locally. To validate our implementation, we compared the extracted values to a reference implementation which used locally stored data. The overall workflow is depicted in Figure 1.

The accuracy of the AI algorithm was measured based on DSC (between a board certified neuroradiologist’s manual segmentations and the AI algorithm’s result) and the correctness of the feature extraction was measured by calculating relative difference compared to a reference implementation.

## Results

**FIGURE 2:**
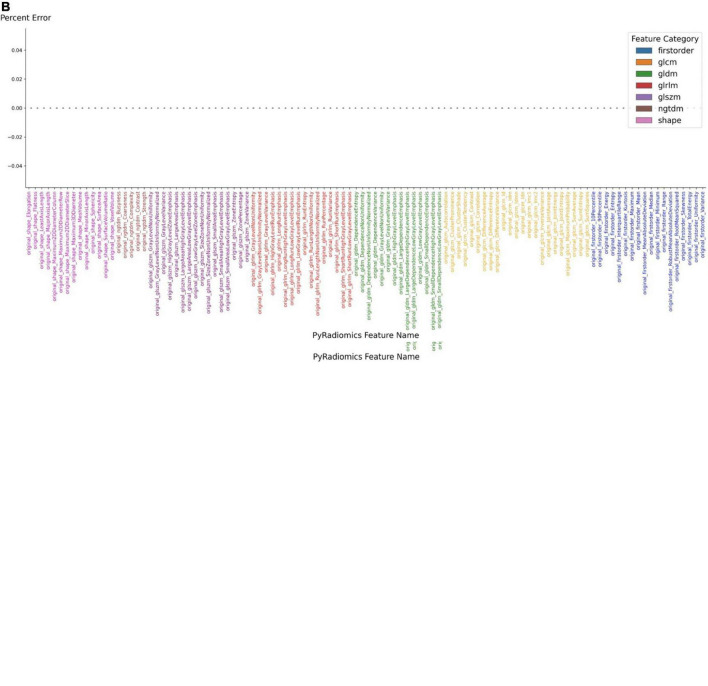
Auto-segmentation tool delineates the borders of tumor and surrounding edema and is similar to manual segmentation of the tumor by neuroradiologist **(A)**. **(B)** Accuracy of segmentation measured using DICE coefficient, Jaccard index, Positive Predictive Value (PPV), True Positive Rate (TPR), and True Negative Rate (TNR).

The UNETR autosegmentation algorithm was trained on FLAIR images from the BraTS 2021 dataset, all 1251 FLAIR series with whole tumor segmentations were used. The training data was split into 1,151 samples for training and 100 for validation. The test set only contained data from YNNH, which was 73 pathologically proven high grade gliomas. Training was stopped after 250 epochs and the model with the best mean DSC (0.90) on the validation dataset was chosen. The model stopped improving after the 170 epoch. On the test dataset, the DSC was lower, but the algorithm still provided a solid baseline segmentation with median DSC of 0.86 and a mean of 0.81. A sample segmentation is depicted in Figure 2.

The developed model was deployed using the NVIDIA Triton inference platform and integrated into the Visage AI Accelerator. The average overall processing time after activating the segmentation algorithm was 2.33 s on our system (NVIDIA TITAN RTX, Intel(R) Core(TM) i7-6900K CPU @ 3.20GHz, 64 GB RAM).

Interaction between the PACS and the algorithm, including image retrieval and storing the resulting segmentation took 0.33 seconds, preprocessing and postprocessing (including brain extraction, image reorientation and resampling) took 1.48 s and the actual inference of the AI model took 0.52 s. Afterward, the segmentation is displayed and modifiable within the client workstation using the same, familiar tools available on the diagnostic workstation.

**FIGURE 3:**
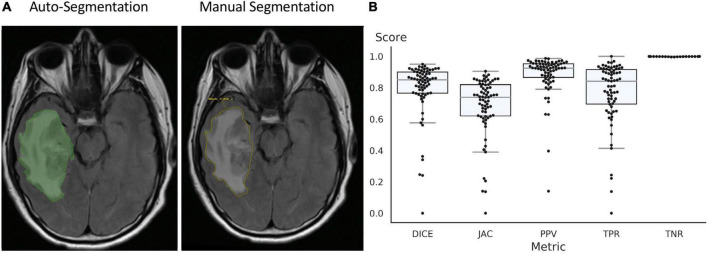
Accuracy of feature extraction using PyRadiomics. **(A)** Features extracted using PyRadiomics incorporated into PACS were compared to features extracted using external PyRadiomic script. The version of the PyRadiomics were v3.0 and v3.0.post3.dev0+ge100f1d. **(B)** The error was brought to zero after the versions of PyRadiomics were matched.

The extraction of radiomic features is then started from the same interface using the embedded PyRadiomics module, with an average computation time of 4 s. The extracted radiomic features can then be exported as JSON files. To confirm the stability of the embedded feature extraction algorithm, it was compared to a reference implementation (EK, TZ). Initially, the features that were extracted from the demonstrated up to 1.4% difference in absolute values with 17 features being different among 106 features total. After inspecting each implementation, a minor version difference was identified. Using the same version (3.0) of the PyRadiomics module resulted in complete agreement, bringing the absolute error to zero (Figure 3). After confirming the correct implementation of PACS based feature extraction, we also performed separate feature extraction on three different time points and found zero error among those separate extraction steps.

Overall, the integration of the glioma segmentation algorithm and feature extraction algorithm into the standard PACS viewer resulted in a workflow efficient and interactive way for users to generate datasets of annotated medical images and corresponding radiomic data. The combination of the image viewer’s standard tools and the availability of algorithms in the form of a button makes advanced quantitative image volume analysis much more accessible in clinical practice.

## Discussion

In this work, we demonstrate that implementation of AI tools into PACS is feasible and provides tools for annotation of medical images and extraction of quantitative imaging features to radiologists in a format that is the same as the clinical workflow. An AI-based workflow including auto-segmentation and radiomic feature-extraction was integrated into a PACS platform and made available to clinicians. The radiologists can use the same PACS interface that they use in clinical practice, thus not requiring additional training or data export for image analysis with ML algorithms. The set of tools interacts with the content visible in the clinical viewer and produces annotations which can be modified using existing tools. The workflow is fast with a total computation time of below five seconds for running both the automatic AI glioma 3D segmentation and radiomic feature extraction tools and easily accessible through buttons integrated into the workstation’s interface.

The quality of the automatically generated whole tumor segmentations from the FLAIR sequence is good with a median DSC 0.86 measured using gold standard segmentations performed by a board certified neuroradiologist. The tool produced quick baseline segmentations which can be approved or modified by the clinician and subsequently is stored in the PACS. The overall result is a fast and interactive pipeline of PACS-integrated tumor segmentation and feature extraction, which is available to the clinician immediately after opening a study. The workflow uses familiar tools and does not require any additional software or coding knowledge. While higher DSC scores were reported in the RSNA ASNR MICCAI BRATS glioma segmentation challenge, our dataset is different due to inclusion of motion degraded but clinically relevant images. Future work will include implementation of advanced algorithms, such as nnUNET which show higher DSC scores.

Currently machine learning in medical imaging is a hot topic with a large spike in publications starting in 2017 (Subramanian et al., 2021; Afridi et al., 2022; Avery et al., 2022; Bahar et al., 2022; Cassinelli Petersen et al., 2022; Jekel et al., 2022). Multiple works demonstrated high accuracy with use of ML for tumor segmentation, identification of images with brain tumors from other pathologies, glioma grade and molecular subtype prediction, differentiation of gliomas from lymphoma or brain metastases. These results suggest that clinical implementation of these algorithms is imminent and will be seen in the clinical practice in the next few years (Subramanian et al., 2021; Afridi et al., 2022; Avery et al., 2022; Bahar et al., 2022; Cassinelli Petersen et al., 2022; Jekel et al., 2022; Tillmanns et al., 2022). The next frontier in neuro-oncology imaging is identification of clinical applications of ML algorithms in clinical practice and determining the aspects of clinical care that can be improved with predictions that can be generated by these algorithms. The best method to achieve adoption, is to bring the ML tools into the hands of everyday radiologists who may or may not have the expertise in computer science or ML, but have extensive expertise in manipulating clinical PACS. PACS has rapidly evolved since its implementation into clinical practice in early 2000s. It has changed the face of clinical practice for radiologists and significantly affected how radiologists interact with the referring providers and hospital staff. The toolset we present in our paper is the first step toward this new reality in clinical practice and will serve as the platform for data curation, annotation/labeling, and AI algorithm implementation.

In addition to enable AI-based interactive workflows, another goal is to develop AI which can run unsupervised on each eligible study. While in most cases AI is not there yet, the persistent and safe storage of segmentations, imaging and clinical parameters in the PACS system enables building of large libraries of datasets. The work in our laboratory is underway to expand the training to additional studies from our institution. Those kinds of datasets accelerate the process of developing and validating such models. The additional information could also already be leveraged for patient management, such as tracking of individual lesions with existing segmentations (Kickingereder et al., 2019).

Our paper has several limitations, which include focus on FLAIR based segmentations for accuracy assessment. The algorithm was originally trained on a BraTS dataset but tested on our single institution data that consists of multiple individual hospitals. The small dataset has been expanded to a larger glioma dataset of 430 cases for development of clinical applications of these tools at our hospital. Another limitation of our study is use of only one feature extraction method (PyRadiomics), which is one of the many methods available (Zwanenburg et al., 2020). The strength of our system is that other feature extraction algorithms can be incorporated in our system, based on the needs of the algorithm that is being used and clinical service where it is being incorporated into.

We present a novel method for implementation of AI algorithms into clinical practice by incorporating a deep learning algorithm for glioma segmentation and radiomic feature extraction into clinical PACS. We validated this method on a single institutional glioma dataset and demonstrate reproducibility of feature extraction. This work has potential for accelerating the translation of research concepts to routine clinical use.

## Data availability statement

The raw data supporting the conclusions of this article will be made available by the authors, without undue reservation.

## Ethics statement

The studies involving human participants were reviewed and approved by Yale IRB. Written informed consent from the participants’ legal guardian/next of kin was not required to participate in this study in accordance with the national legislation and the institutional requirements.

## Author contributions

MA, ML, MW, KB, IT, and AjM contributed to conception and design of the study. SM, GC, RB, HS, PS, ES, and AmM organized the database. KB, WH, ML, and MW developed the PACS platform. KB, TZ, and EK performed the statistical analysis. MA wrote the first draft of the manuscript. MA, KB, EK, and ML wrote sections of the manuscript. JB and MZ contributed to the Yale IT specifications details. All authors contributed to manuscript revision, read, and approved the submitted version.

## Supplementary material

Supplementary Figure 1 Segmentations performed on Visage Software with whole, core, and necrotic portions of the tumor segmented. The whole segmentation **(A)** included tumor core and surrounding edema and infiltrating tumor. The core **(B)** segmentation included the outer margin of the enhancing component of the tumor. The necrotic **(C)** segmentation included the internal necrotic component of the enhancing portion of the tumor.
