# Supplementary figures and images for: Clinical implementation of artificial intelligence in neuroradiology with development of a novel workflow-efficient picture archiving and communication system-based automated brain tumor segmentation and radiomic feature extraction
Source: Front Neurosci. 2022 Oct 13;16:860208. doi: 10.3389/fnins.2022.860208 (PMC9606757; doi:10.3389/fnins.2022.860208)

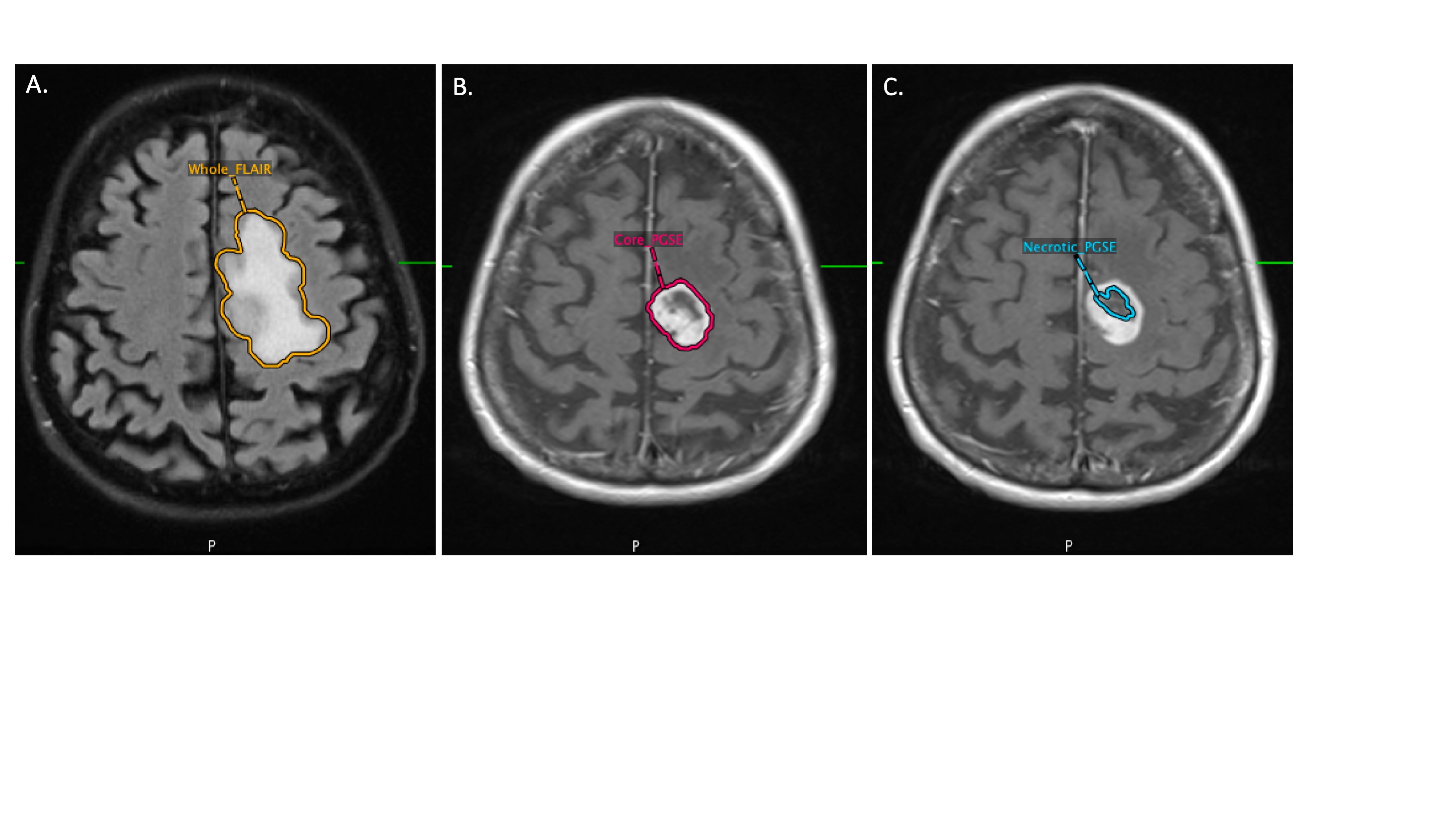

Supplement: Supplementary Figure 1 — Segmentations performed on Visage Software with whole, core, and necrotic portions of the tumor segmented. The whole segmentation (A) included tumor core and surrounding edema and infiltrating tumor. The core (B) segmentation included the outer margin of the enhancing component of the tumor. The necrotic (C) segmentation included the internal necrotic component of the enhancing portion of the tumor. [file Image_1.JPEG]
